# Revisiting patient expectations and experiences of antibiotics in an era of antimicrobial resistance: Qualitative study

**DOI:** 10.1111/hex.13102

**Published:** 2020-07-14

**Authors:** Olga Boiko, Martin C. Gulliford, Caroline Burgess

**Affiliations:** ^1^ School of Population Health and Environmental Sciences King’s College London London UK; ^2^ National Institute for Health Research Biomedical Research Centre at Guy’s and St Thomas’ National Health Service Foundation Trust London UK

**Keywords:** antibiotic prescribing, antimicrobial resistance, patient expectations, patient experiences

## Abstract

**Objective:**

To investigate contemporary patient expectations and experiences of antibiotic prescribing in England.

**Background:**

Primary care providers’ compliance with patient influences has been identified as a motivation for antibiotic‐prescribing behaviour. Since 2013, there have been concerted efforts to publicize and address the growing threat of antimicrobial resistance. A fresh qualitative insight into patient expectations and experiences is needed.

**Design:**

Qualitative study using semi‐structured interviews.

**Setting and participants:**

Two English regions, one an urban metropolitan area and the other a town in rural England. Patients who recently consulted for infections were recruited. The information power approach was used to determine the number of participants, yielding a sample of 31 participants.

**Main measures:**

Thematic analysis was carried out to analyse the interview data.

**Results:**

Five themes were identified: beliefs, expectations, experiences of taking antibiotic, experience of antimicrobial resistance and side‐effects, and experiences of consultations. The accounts** **reflected improved public knowledge: antibiotics were perceived to be much‐needed medicines that should be prescribed when appropriate. The data showed that patients formed expectations of expectations, trying to read the prescribers’ intentions and reflect on the dependency between what prescribers and patients wanted. Patient experiences featured as nuanced and detailed with knowledge of AMR and side‐effects of antibiotics in the context of positive consultation experiences.

**Conclusions:**

The study highlighted complex interplays between adherence to antibiotics and consuming antibiotics in reflexive, informed ways. Ensuring that present and future patients are informed about potential benefits and harms of antibiotic use will contribute to future antimicrobial stewardship.

## 1 BACKGROUND

Public awareness of antibiotic resistance and the need for more judicious use of antibiotics is increasing, but inappropriate use of antibiotics remains widespread.^1^, ^2^ Older studies have ascribed a prominent role to patient influences on antibiotic prescribing, with many studies stressing the view that prescribers may be responsive to patient expectations for antibiotic treatment.^3^ This ‘patient influence’ factor has been identified in most systematic reviews^3^ Estimates from patient surveys suggest that patients’ positive expectations for antibiotics are substantial but have varied between studies.^4^, ^5^, ^6^, ^7^, ^8^, ^9^, ^10^ Family physicians may assume that patients consulting for infections want antibiotics^11^ but primary care clinicians can overestimate the extent to which patients are seeking and expecting antibiotic prescriptions,^11^, ^12^ especially for parents of young children.^13^ There is consistent evidence that GPs are more likely to prescribe antibiotics when their patients are perceived to be expecting them.^8^, ^14^, ^15^, ^16^ A systematic review found a generally positive association between physician perceptions of patient expectation and antibiotic prescription,^17^ but some studies find evidence of a negative association between expectation and prescription^4^ with evidence of inconsistency between physicians’ perceptions and patients’ desire for antibiotics. It is also well established that prescribing antibiotics increases the likelihood that patients will consult in future illness episodes,^18^ raising the possibility that expectations are a consequence not a cause of antibiotic prescribing.

Relationships between patients and primary care providers play a major part in antibiotic‐seeking and antibiotic‐prescribing behaviours. A qualitative study in the UK found that doctors prescribed antibiotics in order to maintain good relationships with patients, with potential patient benefits outweighing the less tangible community risks from antimicrobial resistance.^15^ However, patient expectations are seldom made explicit during consultations. While a high proportion of patients may want antibiotics and expect to be given a prescription, only a minority ask directly for antibiotics.^14^ Some studies confirm that meeting patient expectations is associated with greater patient satisfaction, but other research suggests a more nuanced interpretation. A mixed method study in Australia demonstrated that even though parents consulting with their children wanted antibiotics, satisfaction with their GP visit was not dependent on solely receiving antibiotics.^10^ In a qualitative study of parents consulting GPs in four European countries, parents’ accounts revealed that a trusting and open relationship with the clinician, in which parents felt comfortable to ask questions, challenge and discuss decisions, led them to feel generally satisfied with consultations and accept clinicians’ decisions whether to prescribe antibiotics or not.^19^

In recent years, there have been concerted efforts from scientists, clinicians and policy‐makers to publicize and address the growing threat of antimicrobial resistance both in the UK and worldwide.^20^, ^21^, ^22^ In this context, it is timely to revisit patient beliefs, expectations and experiences of antibiotics and of antimicrobial resistance. Recent systematic reviews have included studies which may antedate current increased concerns for antimicrobial resistance.^14^, ^15^ This study aims to address a need for additional qualitative investigation to understand contemporary patient perspectives on antibiotic prescribing in this era of antimicrobial resistance.

## 2 METHODS

### 2.1 Research design

The aim of the current study was to examine contemporary patient expectations and experiences of antibiotic prescribing in England. The study was approved by the London‐Hampstead NHS Research Ethics Committee 18/LO/1874. Where applicable, the study followed the Consolidated Criteria for Reporting Qualitative Research (COREQ) checklist for reporting qualitative research.^23^

Semi‐structured interviews were conducted with patients who consulted general practice for infection in two English regions, one an urban metropolitan area and the other a town in a rural part of England with a high demand for primary care services. The two regions represented diverse contexts for general medical practice. The participants were invited to be interviewed if they had recently consulted and been diagnosed by a GP as having a bacterial infection. The bacterial infections were identified using Read codes for the relevant conditions including respiratory tract infections, urinary tract infections and skin infections as the major indications for antibiotic prescribing. An interview guide was developed to reflect expectational structures associated with antibiotics as well as the experiences of illness and experiences of consultations. The items were informed by a review of the literature and included past and current experience of being prescribed antibiotics, knowledge, beliefs and attitudes towards taking antibiotics and interactions with medical practitioners. The questions were discussed among the research team and piloted with a small number of patients before refining. The items in the topic guide were organized under six main headings (Table 1). All interviews were conducted by the first author (OB) to ensure consistent quality. The interviewer has a PhD in medical sociology and is an experienced qualitative researcher. Interviews were conducted in the period February‐December 2019. Interviews lasted between 13 minutes and 42 minutes.

**Table 1:** Patient interview schedule

1. Experience of medical consultation To begin with, could you tell me about your recent experience of consulting the GP for infection? What was the health issue and how it was dealt with? Did you have any expectations of specific treatment? Were you able to discuss them in the consultation? Was the risk associated with different treatment choices communicated and how? How was the issue resolved? Has seeing the doctor helped in infection management?
2. Knowledge of antibiotics Overall, what is your knowledge about different types of infections and associated treatment? Could you share with me what is your understanding of antibiotic treatment? How do the antibiotics work? What types of antibiotics are there? When should antibiotics be prescribed? What are the risks associated with non‐prescribing of antibiotics? What are the potential complications and unwanted consequences of AB treatment? Who should be making decision on AB treatment? What was your previous experience of AB treatment, if any? To what extent your experience shaped your perception of antibiotics at present? Were there any changes in how you consider infections and their treatment? What has driven these changes?
3. Concerns about treatment Would you say that you felt confident in managing the infection with/without treatment? Were you able to raise your concerns and have all your questions answered during the consultation? Have you experienced any difficulties in complying with the treatment plan?
4. Optimism regarding outcomes Are you hopeful for the AB treatment to be the best possible course of action? If there was an uncertainty and anxiety around the treatment plan, how did you handle it? Have you been able to seize the impact of AB treatment following the recent or previous consultations for infections?
5. Decision‐making processes What would be your priorities in infection management? In consultations for infections, if a doctor's advice differed from your interpretation, would you or have you challenged the decision? What would be/were your actions following unresolved or repeated infection?
6. Social and environmental influences Speaking about the appropriate treatment for infections, what are the sources of information that are likely to influence your understanding? In your experience, are doctors consistent in their consultations for infections? What your friends, family members and close networks believe with respect to AB treatment and how it compares with your beliefs? What are your perceptions of antimicrobial resistance? What other information might be useful in making decisions on antibiotics treatment?

### 2.2 Recruitment of participants and data collection

Metropolitan practices were invited to the study by the local Clinical Research Network who generated the expression of interest; a practice in a town in a rural area of England was recruited through informal Clinical Research Network contact. The researcher's invitations to take part generated expressions of interests from the general practices that agreed to purposively select patients who visited a primary care professional for infection in the last 6 months. Patient lists were approved by a general practitioner acting as research gatekeeper in each practice and initially 927 patients were sent invitations to study via the Docmail postal system. The invitations contained a letter from the practice inviting patients to take part and information sheet. Patients who agreed to take part either returned reply slips or contacted the researcher using the contact details provided. The researcher then communicated via email or text message to establish the contact followed by sending the consent form and confirmation of the interview meeting.

In total, 33 patients agreed to participate. The interviews with two patients were discarded: one involving a parent interview and in another where the patient did not consult for an infection. Out of 31 patients (Table 2) who comprised the final sample, 26 patients were interviewed face‐to‐face in patients’ homes and five patients were interviewed via phone (see Table 2). The sample size was determined using the pragmatic concept of ‘information power’,^24^ which proposes that the size of a sample with sufficient information power depends on (a) the aim of the study, (b) sample specificity, (c) use of established theory, (d) quality of dialogue and (e) analysis strategy. While our aim was (a) broad and (b) specificity is biased towards one group (almost half of the interviewees were older female patients consulted for urinary tract infection), we followed (c) a theoretical model to explain the findings and (d) the quality of the interviews is relatively high. Since we aimed for (e) a cross‐case analysis, we decided to continue recruitment until the sample size reached thirty‐one eligible patients.

**Table 2:** Participant characteristics

Characteristic	Value	Frequency
Age (years)	20‐29	2
	30‐39	3
	40‐49	4
	50‐59	3
	60‐69	4
	70‐79	9
	80‐89	5
	90‐99	1
Gender	Female	24
	Male	7
Ethnicity	White (British)	25
	White (Other)	3
	Black	2
	Asian	1
Indices of multiple deprivation	High	12
	Medium	9
	Low	8
Region	Urban	22
	Shire‐town	9
Type of infection	Respiratory	16
	Urinary	11
	Skin	4

### 2.3 Analysis

The interviews were digitally recorded, transcribed verbatim by a professional transcriber, imported to an NVivo‐12 project and coded through an iterative six phased process described in thematic analysis.^25^ Data analysis occurred iteratively and involved familiarization, coding, theme searching, theme reviewing, theme defining and naming, producing the report. Repeated patterns in the data formed the basis for the codes, identified by the first author, and one single code for every different concept/idea was generated. To ensure that codes were applied consistently, a co‐author (initials) independently coded a random sample of four interview transcripts. Coding was refined after discussion. Data identified by the same code were collated together, and all different codes were sorted into potential subthemes and themes using NVivo options of tree building. Then, the potential themes were re‐assessed and re‐organized to reflect major narratives and themes in the coded data. Finally, the authors refined and named the five main themes and subthemes. Participants’ feedback on the transcripts or the summarized final findings was not sought. This research was part of a larger project concerning the safety of reduced antibiotic prescribing. The research is supported by a patient and public involvement group, including eight members of diverse ages and ethnic origins. The process of developing subthemes and themes was discussed at a patient and public involvement meeting, and feedback received was included in the interpretation.

## 3 RESULTS

We summarize the results under the headings of five main themes that were identified in the thematic analysis. Analysis did not identify systematic differences according to metropolitan or rural location nor according to mode of interview completion.

### 3.1 Beliefs about antibiotics and antimicrobial resistance

Antibiotics emerged as trusted medicines that had widespread use; the descriptors used by the interviewed patients ranged from ‘magic answer’ to ‘sledgehammer’ treatment. Those who referred to antibiotics as a magic pill were often older females with recurrent urinary tract infections with expectations for apparently appropriate prescribing. On a whole, the most common belief among the interviewees was that antibiotics should be prescribed and taken when necessary. The patients’ concerns were rather about ‘*finding the right one for what infection you have at the time and making sure that you're going to be safe’* (Int 13, *female, chest infection*). There was also recognition of the need for better scrutiny in prescribing, which highlighted the complexity of decision making concerning safe antibiotic treatment.

The interviewees spoke about different thresholds of illness and about reducing use of antibiotics. There were accounts of decreased impact of antibiotic and of antimicrobial resistance by informed patients, especially by younger, and more educated patients:

I: Tell me more about antibiotics. What do you know about antibiotics?

R: the effectiveness of some of our standard treatments is decreasing rapidly. And I believe that a lot of that is to do with inappropriate usage, both inappropriate prescribing which I have witnessed myself. But then also inappropriate use by patients of antibiotics. (Int 20, female, tonsillitis).

Or in another example:

I: Do you want to add anything else in terms of your experience with antibiotics?

R: I find it alarming that we may be getting towards the end of the road with antibiotics…They're finding ways of compensating for the overuse of antibiotics. And clearly there are issues with the pharmaceutical industry as to how much they're prepared to invest in developing new antibiotics… And I don't want to get political about it but there needs to be some sort of disentanglement of the profit motive, which I understand and the service to the general public. (Int 4, male, urinary tract infection)

### 3.2 Expectations of antibiotics

Interviewees’ expectations varied: approximately half of the interviewees expressed a wish for an antibiotic, but the other half had undifferentiated expectations of help in getting better when attending for the consultation. Those with recurrent urinary infections identified wanting antibiotics based on their previous experiences:

I: Did you have expectations of specific treatments when you went to see the GP?

R: While I was waiting for the antibiotics, I also tried stuff myself from the chemist. And also, drunk the cranberry juice, which was no good. I knew that I needed antibiotics. It seems for me if I get cystitis it starts, and it comes on really quickly. (Int 12, female, urinary tract infection).

Those with chest infections had thought about the need for antibiotics but preferred to leave the decision concerning antibiotic treatment to health‐care professionals:

I: When you went to see the doctor, did you expect a particular treatment or prescriptions?

R: I did expect that if it is something on the lungs, that I would be given antibiotics. But I didn't push for them or anything. I really went there to, to see what it is. But I wasn't particularly surprised that they heard the noise on the lungs. (Int 18, male, chest infection)

Patients interviewed often referred to antibiotics as something that would ‘shift’ their illness, but also as a symptomatic cure and something ‘to boost the immune system with’. This female patient held a radically different view:

I: Okay, so, you didn't expect any particular treatments or antibiotics in particular?

R: I feel bad to say this to the doctor, but I’m quite anti the use of antibiotics. I think I’ve read too many horror stories about overuse of antibiotics. So, I avoid them at all costs. And I definitely wouldn't have even considered antibiotics unless the doctor had mentioned them. (Int 20, female, skin infection)

Approximately half of the interviewees, and those who were not consulting for urinary tract infection, had less differentiated expectations:

I: Did you have any expectations of specific treatment when you came with the symptoms?

R: I didn't have any expectation of treatment. And, in actual fact, I remember thinking, ‘Oh do I really need antibiotics?’ But I don't know, I guess with kind of almost pre‐programmed to say, ‘Okay, if that's what you want to give me, that's what I’ll take’. So, I didn't really have any expectations. To be honest, I hadn't even considered that I may have tonsillitis. (Int 31, male, tonsillitis)

In terms of how interviewees perceived prescribers’ expectations, there was a difference in understanding of the prescribers’ mindset. Several interviewees held a view that prescribers’ expectations were contingent on patient expectations so that they brought up an association with patient pressure: ‘*…they don't want to prescribe antibiotics till they see that you are really, really desperate or you need it’ (Int 6, female, tonsillitis)*. Prescribers’ expectations were in these cases dependent on patients’ wishes as well as on symptoms. Others believed that patient expectations might not influence the professional's decision, which might only depend on clinical findings. Such view was based on critical reflection, for example:

I: What do you think doctor thought of, about your expectations?

R: I don't think a doctor, any GP in the UK takes into consideration a person's expectations. They give, they prescribe based on the symptoms that you present. Not necessarily your expectations. (Int 16, female, urinary tract infection).

### 3.3 Experience of taking antibiotics

The interviewees had different exposure to antibiotics: some of them were prone to recurrent infections and were prescribed antibiotic more than once in recent months, others received an occasional prescription for antibiotic. Most of them described days and weeks of experiencing illness before they consulted a clinician. Apart from one case, there had been a sense of welcoming antibiotic treatment, for example according to a patient with chest infection ‘*antibiotics, without being dramatic, saved my life’ (Int 1)*. At the same time, many patients were reflexive about the role of antibiotic in coping with the ailments. For example, a patient with tonsillitis questioned the appropriateness of prescribing:

I: So, it seems like it cleared everything off? Was it efficient?

R: To be honest, I remember starting to feel like my throat was feeling better within 12 hours of taking the antibiotics. And, at that stage, I started to think, okay, actually maybe I didn't need antibiotics because this has cleared up very quickly and, in hindsight, kind of thinking about it, thinking, well there's no way the antibiotics would work that quickly. (Int 31, male, tonsillitis)

The interviewees had a range of various experiences of past antibiotic treatment. Patients with a history of infectious diseases shared positive accounts of antibiotics in general:

I: So, obviously were you hopeful for the antibiotic treatment to be the best possible course of action when you came with chest infection?

R: I’ve had chest infections before. They've always cleared up and you know I’ve never sort of thought it could lead to anything significantly worse. I’ve always been confident that whatever antibiotic I was given would do the job, you know”. (Int 23, male, chest infection)

### 3.4 Experience of antimicrobial resistance and side‐effects

Approximately one‐fourth of the patients spoke about their experience of antimicrobial resistance. The accounts were full of frustration and confusion because either antibiotics had not worked or the first‐line treatment had not worked. This was especially true for patients with urinary tract infections but also with tonsillitis. Several patients experienced up to three to five episodes of urinary tract infection a year and found it hard to tolerate its recurrent nature. For example,

I: Did you mention you had a bad reaction to that as well?

R: I felt really ill in the morning. I didn't know why, and I had to drive somewhere. And oh, and really, really had to go to bed. I felt so bad. And then on the Monday as I say, I had a call from the surgery to say they'd discovered that it was resistant to that and they changed it… I have heard antibiotics can turn toxic in you if they're not the right one, can't they? (Int 24, female, Urinary tract infection)

Antibiotics were sometimes associated with mild‐to‐serious side‐effects; for example, an anaphylactic allergic reaction was coupled with resistance in the following account:

I: Did you say you were allergic to one particular antibiotic?

R: I was allergic, yes.

I: How did you know that?

R: I was given penicillin that was in 2000 and I had an anaphylactic shock. I was rushed into hospital and I had to spend a night in the emergency room and given steroids for 5 days because of that. So, then I couldn't take penicillin, I couldn't take erythromycin and I became resistant to doxycycline and there were other ones that I started to become resistant to, that didn't work. (Int 13, female, chest infection)

Several interviewees confirmed that the most common side‐effects were nausea, stomach upset, vomiting but also thrush. The interviewees spoke about compensating with probiotics to resolve digestive side‐effects but continued with antibiotics. Where discontinued or prescribed different antibiotic, this was in the case of more significant side‐effects such as liver derangement or shortness of breath like in the following extract:

I: So, have you been, having any side effects because of antibiotics?

R: But when I was prescribed it a second time, within 24 hours I was getting very short of breath… it was clear to me that this was having an effect on my breathing. So, I went straight back to the GP and then they put me on, on a different antibiotic. (Int 4 male, urinary tract infection)

### 3.5 Experience of consultation

The patients interviewed reported predominantly positive experience of consultation with a prescriber they have recently seen for infection. For the exception of very few rushed encounters which did not have a room for asking/answering questions, most consultations were described as patient‐centred:

I: And how did the consultation go?

R: It was good. She (prescriber) was very thorough. She understood that 3 weeks of suffering was quite a long time, so she was understanding. So, yes, she was thorough, she was very sympathetic, and I felt silly going about something that was really, I thought just a cold. But she said she could tell that I was in discomfort and she listened to my symptoms. (Int 2 female, sinusitis)

Meanwhile, the patients were concerned with the issue of appropriateness of antibiotics and understood uncertainty and complexity associated with it. They appreciated both diagnostic uncertainty and how it has been resolved in real‐time consultations. A patient perceived the complexity of clinical decision making against risks for patients’ health:

I: Who should be making decisions on antibiotics, the doctors or the doctors and patients or maybe patients?

R: … and the doctor would have to be able to take a closer look and to really be able to assess whether he can take the risk of not giving the antibiotics or not, because maybe it's, it's just finding a balance of hitting the riskiest bit of this illness first and then dealing with the side effects as you go along”. (Int 18, male, chest infection)

It was also apparent that the patients interviewed reflected on patients’ collective state of mind and role in pressurizing a prescriber: ‘*I’m sure many people lie just to get antibiotics’. (Int 19, female, tonsillitis)*. There were, however, consultations in which shared decision making took the form of expectation elicitation. Reflexivity emerged in these cases where prescribers directly elicited patient expectations. Two patients admitted being overtly asked about their agreement to use antibiotics, for instance:

I: So, to begin with can I ask you what's your recent consultation with GP? What was the problem?

R: … she (prescriber) said to me would I mind taking some antibiotics for that. And I said if she felt that they would help then yes, I did. It was a 5‐day course… Yes, that was the first time actually that I’ve sort of been asked rather than told”. (Int 13, female, chest infection)

## 4 DISCUSSION

### 4.1 Main findings and comparison with the literature

Participants perceived antibiotics to be much‐needed medicines that should be prescribed when appropriate. Expectations for antibiotic treatment were often conditioned on previous experiences. However, past experiences were not restricted to successful treatment of infections but also included experience of antibiotic resistant infections, antibiotic drug side‐effects and inappropriate prescribing. Patients’ views were also informed by genuine concern about antimicrobial resistance. These lay accounts appeared to reflect the contemporary medical ideas of ‘precision’ or ‘personalized’ medicine, which are represented in the slogan of the ‘right drug for the right patient at the right time’.^26^

Consistent with other studies,^9^ participants’ knowledge about side‐effects was not associated with their expectations of antibiotics. The concern about and experience of antimicrobial resistance found in our sample contradicts evidence from a review of general public attitudes which reported low awareness of AMR.^27^ We found that around a quarter of the sample experienced AMR in one form or another and these participants were more sceptical about antibiotics. The accounts of those who experienced AMR were full of frustration and confusion because either antibiotics had not worked or the first‐line treatment had not worked. Likewise, in the qualitative arm of their study,^8^ Gaarslev et al (2016) established a growing number of patients who knew antibiotics did not kill viruses and who agreed that taking antibiotics when not needed means they may not work in the future. Although compliant with antibiotic treatment, participants in our study raised important questions of the right antibiotics being prescribed at the right time. Their accounts of illness suggested explicit and informed choices behind the experiences of both treatment and consultation for infections.

Participants tried to justify the prescription by relating to prescribers’ decision‐making processes, and their own and prescribers’ expectations. Some interviewees believed that prescribers seek to meet patient expectations who, in their turn, acted on an adherence principle. We suggest that adherence is often determined by complementarity of expectations. Although we did not attempt to quantify findings, we established that the rate of patients expecting antibiotics was relatively high (half of the participants); this finding however can be explained by a high proportion of patients consulted for urinary tract infections, an often recurrent health condition requiring appropriate antibiotic treatment. Our data also showed that patients could form expectations of expectations, trying to read the prescribers’ intentions and reflect on the dependency between what prescribers and patients wanted. Patients were also aware of the possibility of patient pressure: a patient acknowledged that other patients’ intentions may be based on exaggeration to receive a prescription. These accounts evidenced the observation that practitioners’ perceptions of patient expectations matter, rather than patient expectations per se.

Where participants were debating the appropriateness of prescribing, it was associated with informed choice and shared decision making. Information sharing is a prerequisite to shared decision making, and it appears that patients want information about their medical condition and treatment options without necessarily being responsible for making treatment decisions.^28^, ^29^ In the medical sociological literature on late modernity, it is argued that individuals experience self, the body and the social and physical worlds with a high degree of reflection, questioning, evaluation and uncertainty.^30^ It is assumed the ‘consumerist’ patient and the ‘reflexive’ actor ‘both are understood as actively calculating, assessing and, if necessary, countering expert knowledge and autonomy with the objective of maximizing the value of services such as health care’.^30^ (p. 134) Recently, it has been shown that when informed about individual and social consequences of antibiotic overuse, patients may be more receptive to antibiotic prescription limits.^31^ This evidence suggested that the patient role involves staying informed about the issue of antibiotic use and considering potential benefits and harms when making decisions about antibiotic use. We found that informed patients (of antibiotics and the associated risks) displayed more satisfaction with the consultation. An interview study in Australia^32^ also established that most consumers would accept the GP’s decision not to prescribe an antibiotic if it was clearly explained. Therefore, the re‐emergence of the informed patient is inevitable in the era of antimicrobial resistance, where expert knowledge of antibiotics is broadcast through the media and public health campaigns.

The recent research demonstrated that patients were unwilling to follow the prescriber's recommendations blindly and wanted to know about appropriateness of prescribing^12^ and our study of expectations and experiences lent support to this. Patients seemed to have been more prepared to openly deliberate on prescribing decisions and their expectations were more explicit than previously, even though trust in clinician still had a major role to play.^12^, ^19^, ^33^ Those participants who emerged as informed patients rejected a blind compliance. Indeed, patient expectations were due to disclosure; for example, it was manifested in the consultations that used elicitation (of expectations) technique. Expectation elicitation by clinicians, directly or indirectly (by running commentary), and their open communication appeared important for on‐going clinician‐patient relationship.^11^, ^19^, ^34^, ^35^ Instead of trying to read the patient's mind, prescribers were and must be making the expectations apparent by asking about them. Patients should be making their expectations clear and evidenced by explaining symptoms, which commonly convenience medical professionals.^36^, ^37^ This should be done in contrast to patient demand as it is threatening clinical autonomy.^38^

### 4.2 Strengths and limitations

We established the variety of patients’ expectations, which in some cases attested to an unquestionable compliance and in other cases—to reflexive accounts of expectations of expectations. A wish for the right antibiotic with no resistance and no side‐effects prescribed at the right time (for bacterial infection) confirmed the patients’ expectations of appropriate treatment. This paramount expectation, according to the patients interviewed, was actualized in consultations with prescribers. Moreover, the patient experiences appeared more nuanced with more knowledge of AMR and side‐effects of antibiotics than might be assumed. Prescribers might be reassured that their patients may be knowledgeable and accepting of the limitations of antibiotics.

While we investigated the accounts of patients, we could not triangulate our analysis with the account of health‐care professionals, which makes our analysis one‐sided. Similarly, our aim was not in observing actual encounters but rather examining the expectational structures of patients. Some qualitative research endeavoured to compare the perspectives of professionals and patients (eg,^4^) and questioned the pressure originating from patient expectations. Our analysis demonstrated that the participants believed that patient expectations did not influence prescription, in half of encounters because the antibiotic treatment was evident (urinary tract infections) and in the other half—down to the fact that expectations were undifferentiated. However, because we did not have the prescribers’ accounts we could not draw conclusions about the actual impact of patient expectations. Ideally, expectations on both sides should be studied to answer the validity of notion of expectations of expectations. The study was conducted in the context of general practice in England. Although attempts were made to recruit patients based on purposive sampling, the sample of our study was skewed towards White British female patients who consulted for urinary tract infections. This affects the results in the form of patients explicitly expecting antibiotics and their appropriate prescribing. Follow‐up studies should attempt to diversify the sampling by purposefully including patients of different backgrounds consulting for other bacterial infections. For example, interpretation of our data suggested that educational level might be influential. Future studies should also aim to include patients with infections who were not prescribed antibiotics to evaluate their experiences of illness and consultation.

## 5 CONCLUSIONS

While compliant with antibiotic treatment, participants in our study raised important questions concerning the right antibiotics being prescribed at the right time. The accounts of illness suggested explicit and informed choices behind the experiences of both treatment and consultation for infections. Patient experiences featured as nuanced and detailed with knowledge of AMR and side‐effects of antibiotics. A high rate of patients expecting antibiotics can be explained by a high proportion of patients that consulted for urinary tract infections, a recurrent health condition requiring appropriate antibiotic treatment. Our study highlighted complex interplays between adherence to antibiotics and consuming antibiotics in reflexive, informed ways.

These findings offer an important message to health‐care workers who may be involved in prescribing antibiotics in primary care. Patients seeking advice for common infections in primary care may benefit from explanation, and information concerning appropriate treatment options, accounting for risks both from prescribing and withholding antibiotics. Inappropriate or unnecessary antibiotic prescribing is commonplace in primary care, but this no longer appears justifiable in terms of patients’ expectations and knowledge of drug side‐effects and antimicrobial resistance. From a public health perspective, efforts to inform the public and potential patients of the risks of inappropriate antibiotic treatment, as well as the conditions in which timely treatment is required, should be a key element of continuing antimicrobial stewardship efforts.

## CONFLICT OF INTEREST

The authors have no conflicts of interest.

## Data Availability

The data that support the findings of this study are available from the corresponding author upon reasonable request.
